# Community‐based physical activity interventions for adolescents and adults with complex cerebral palsy: A scoping review

**DOI:** 10.1111/dmcn.15611

**Published:** 2023-04-09

**Authors:** Prue Morgan, Stacey Cleary, Iain Dutia, Keegan Bow, Nora Shields

**Affiliations:** ^1^ Department of Physiotherapy Monash University Melbourne Australia; ^2^ Neurodisability and Rehabilitation Murdoch Children's Research Institute Parkville Australia; ^3^ Department of Paediatrics, Faculty of Medicine, Dentistry and Health Sciences University of Melbourne Parkville Australia; ^4^ Physiotherapy, Faculty of Health Sciences Australian Catholic University Brisbane Australia; ^5^ School of Human Movement and Nutrition Sciences The University of Queensland Brisbane Australia; ^6^ Physiotherapy, Klint Neuro Forest Hill Australia; ^7^ Olga Tennison Autism Research Centre, School of Psychology and Public Health La Trobe University Melbourne Australia

## Abstract

**Aim:**

To identify implementation strategies and safety outcomes (adverse events) of community‐based physical activity interventions for adolescents and adults with complex cerebral palsy (CP).

**Method:**

Five electronic databases were systematically searched to April 2022. Data were extracted on the implementation and safety of physical activity interventions for adolescents and adults with CP, classified in Gross Motor Function Classification System (GMFCS) levels IV and V, delivered in a community setting.

**Results:**

Seventeen studies with 262 participants (160 participants classified in GMFCS levels IV or V) were included. Community settings included schools (*n* = 4), participants' homes (*n* = 3), gymnasia (*n* = 2), swimming pools (*n* = 2), and other settings (*n* = 4). Most studies specified medical or safety exclusion criteria. Implementation strategies included pre‐exercise screening, use of adapted equipment, familiarization sessions, supervision, physical assistance, and physiological monitoring. Attendance was high and attrition low. Nine studies reported non‐serious, expected, and related events. Four studies reported minor soreness and four studies reported minor fatigue post‐exercise. Serious adverse events related to exercise were infrequent (reported for 4 of 160 participants [<2%]: three participants withdrew from an exercise programme and one participant ceased exercise for a short period). Most frequently reported was pain, requiring temporary exercise cessation or programme change, or study withdrawal (three participants).

**Interpretation:**

For most adolescents and adults with CP classified in GMFCS levels IV and V, physical activity interventions can be safely performed in a community setting, without post‐exercise pain or fatigue, or serious adverse events.

**What this paper adds:**

- Supervised community‐based physical activity interventions can be safely performed by people with complex cerebral palsy.
- Post‐exercise pain or fatigue was not common among those classified in Gross Motor Function Classification System levels IV or V.
- Serious adverse events are infrequent when exercising in community settings, with safety strategies.

## What this paper adds

- Supervised community‐based physical activity interventions can be safely performed by people with complex cerebral palsy.
- Post‐exercise pain or fatigue was not common among those classified in Gross Motor Function Classification System levels IV or V.
- Serious adverse events are infrequent when exercising in community settings, with safety strategies.

Video Podcast: https://youtu.be/QzHoeTYOOsU

Adolescents and adults with cerebral palsy (CP) are less physically active and spend more time sedentary than their typically developing peers.^1^ This is associated with greater risks for physical, metabolic, and social ill‐health.^1^ Being physically active as an adolescent with CP doubles the probability of being physically active as an adult,^2^ which may reduce the risk of ill‐health in adulthood. A systematic review of 49 studies exploring the impact of leisure time physical activity for children and adults with CP reported improved health, fitness, and physical function outcomes following a range of physical activity interventions.^3^ However, the available evidence was gleaned primarily from ambulant children (those classified in Gross Motor Function Classification System [GMFCS] levels I–III).^4^ Adults, wheelchair users, and those with motor function classified in GMFCS levels IV and V were vastly under‐represented,^3^ with only seven of the 49 reviewed studies including adults, and only 5% of the 1513 participants having gross motor classifications in GMFCS levels IV or V.

Given the substantial lifespan effects of inactivity on motor, metabolic, cognitive, and psychosocial health, it is important that appropriate physical activities can be accessed safely by people with CP of all ages and complexities. Adults with CP classified in GMFCS levels IV and V are at even greater risk of chronic health conditions such as cardiovascular disease and diabetes mellitus,^5^ and frequently experience premature mobility decline.^6^ It is therefore imperative to identify suitable physical activity interventions in settings acceptable with this group, to address their significant health needs.

A recent mixed‐methods systematic review synthesized the barriers and facilitators of physical activity participation for adolescents and adults with childhood‐onset physical disability.^7^ The authors concluded that participating in physical activity was enhanced through positive social connections, social support, and an appropriate and accessible physical environment.^7^ However, it was noted people with physical disabilities expressed doubts about their capabilities, fear of injury and pain, concerns about the energy they require to be physically active, and the impact of exercise on fatigue.^7^ Unsurprisingly, those with more complex physical disabilities, such as people with CP classified in GMFCS levels IV and V, described not engaging in physical activity because of their own concerns around their safety and health, or because of the concerns around perceived fragility expressed by their immediate social networks or other stakeholders (e.g. family, personal trainers). Health professionals have also acknowledged fear as a barrier to physical activity participation in adults with disability,^8^ which can perpetuate the perception that people with severe disability should not participate in physical activity owing to unspecified risk. While all physical activity incurs some risk, identification of the nature of any risk, and potential strategies to ameliorate such risk, would be useful to enhance physical activity opportunities and engagement for people with complex CP.

Key to physical activity participation for young people with disability are inclusion and involvement in the community.^7^ For people with disabilities, this fosters positive mental health, enhances physical health benefits (such as optimizing functional independence), provides opportunities for social relationships, and improves quality of life. Furthermore, young adults with CP express a desire to move away from engaging with therapy interventions in a clinical environment^9^ to participating in non‐healthcare settings, to maximize their social interactions and facilitate social connectedness. However, ensuring safety while exercising in community settings for those with complex disability is important to counter fear and perceptions of fragility. Understanding the initiatives that can be used to support people with complex forms of CP to participate safely in community‐based physical activity interventions can inform best practice. This will lead to better health outcomes and increased community‐based physical activity participation for those with complex CP. The aims of this scoping review were therefore to identify implementation strategies and safety outcomes (i.e. presence or not of adverse events) following community‐based physical activity interventions for adolescents and adults with complex CP.

## METHOD

This scoping review was guided by the methodological framework proposed by Arksey and O'Malley,^10^ the key stages of which are (1) identification of the research question; (2) identification of relevant studies; (3) study selection; (4) data charting; and (5) collation, summarizing, and reporting of results. This protocol was registered with the Open Science Framework on 26th May 2022 and is reported here according to the PRISMA Extension for Scoping Reviews guidelines.^11^

### Eligibility criteria

Eligibility criteria were (1) studies that investigated the implementation of physical activity interventions for adolescents and adults (mean age 10 years or older); (2) a diagnosis of CP; (3) classified in GMFCS levels IV and V; (4) delivered in a community setting (i.e. not in a health or research facility); (5) all languages; and (6) peer reviewed. The mean age threshold was chosen because adolescence – the phase of life between childhood and adulthood – starts from age 10 years.^12^ Physical activity interventions may have included any programme designed to progressively improve any aspect of physical function (including gait and balance), with a measure of physical activity or physical fitness (including strength, fitness/aerobic capacity) included as an outcome. Programmes that focused exclusively on improvement of upper limb dexterity or cognitive function were excluded. If studies included participants across a range of disability diagnoses, the proportion or number of participants with CP and specific data referring to this subgroup needed to be reported separately. Similarly, if the study included participants with a range of gross motor abilities (i.e. broader than GMFCS levels IV or V), the specific number of participants with CP classified in GMFCS levels IV and V must have been able to be identified. All types of quantitative study design were eligible for inclusion, as the aims of this review related to safety and intervention implementation rather than efficacy. Both experimental and quasi‐experimental study designs, including randomized and non‐randomized controlled trials, before and after studies, prospective and retrospective cohort studies, cross‐sectional, and descriptive observational study designs were considered. Studies were included if they were completed in a public or private community setting, or in a participant's home. Interventions supported by a health professional, trainer, teacher, or family member were considered, as the focus of the review was on the safety of being active or exercising in the community, not on the qualifications of the supervisor.

### Search strategy

Following consultation with a subject‐matter librarian, the following databases were identified and searched from inception until 30th April 2022: PubMed (since 2021 only), Medline, Embase, CINAHL Plus, and AMED. Medline is the largest contributor to PubMed, meaning that any articles identified by PubMed would also be picked up by the Medline search; therefore PubMed was searched from 2021 only to ensure citations that preceded the article's final publication in a Medline indexed journal were identified. The search strategy included a comprehensive list of keywords, MeSH headings, and derivatives relating to the concepts of CP and physical activity (Appendix S1). The electronic searches were supplemented by citation tracking of included studies using Google Scholar and reference list checking of final included articles.

### Selection of sources of evidence

The search yields were combined, duplicates removed, and the remaining items uploaded into Covidence (Veritas Health Innovation, Australia) for screening. Two reviewers independently screened the studies from the initial yield, on the basis of title and abstract, using agreed inclusion and exclusion criteria. If consensus was unable to be agreed upon, the full text was retrieved and reviewed. Articles not written in English were translated using online software (Google Translate). Images of these articles were uploaded, translated, and then full texts were assessed independently for inclusion by two reviewers; any disputes were resolved by discussion. Protocol papers were retrieved or primary authors were contacted to clarify information about inclusion criteria when necessary. Reasons for exclusion of sources of evidence at full‐text stage were recorded and reported.

### Data extraction and charting

A standardized spreadsheet was developed and piloted for use in the review to gather information about study design and participants' details (country of origin, study type, study location, sample size, mean age, sex, GMFCS level). If GMFCS levels were not explicitly reported, GMFCS equivalence was estimated from clinical descriptors (e.g. non‐ambulant was considered equivalent to GMFCS levels IV or V). A modified TIDieR (Template for Intervention Description and Replication) checklist^13^ was used to guide data extraction on intervention, procedures, duration (total minutes of intervention delivered), safety parameters implemented, and any adverse events. Data extraction was completed independently by two reviewers.

### Collation and summarizing of results

The last stage of the Arksey and O'Malley framework,^10^ collation and descriptive synthesis of data, was performed. Organization of relevant findings into themes and categories, particularly with reference to safety and adverse events was performed.

## RESULTS

A systematic search returned 3419 items for screening (Figure S1). Once duplicates were removed and abstracts screened, 85 full‐text articles were assessed for eligibility. Seventeen studies with 262 participants, 124 (47%) females, and 160 (61%) participants with gross motor function classified in GMFCS levels IV or V were included (Table 1). Six of these studies included only participants classified in GMFCS levels IV and V while the remaining 11 studies also included participants with less severe impairments (GMFCS levels I, II, and III) (Table 1). There were 11 studies where participants had a mean age of 18 years or younger; one study where participants had a mean age in young adulthood (20 years); and five studies where participants had mean ages in middle adulthood (range 36–50 years).

**TABLE 1:** Study characteristics and participants' details.

Reference, country	Study design	Study context	Sample size (*n*)	Mean (SD) age and/or range, years:months	Sex (female:male)	GMFCS	Exclusion criteria
Armstrong et al.,^36^ Australia	RCT	Home and tertiary hospital	21 Exp: 11 Con: 10	10:4 Exp: 9:10 Con: 10:10	13:8 Exp 6:5 Con 7:3	II/III/IV 6:6:9 Exp II/III/IV 3:3:5	‐ Contraindications to/unable to tolerate FES ‐ Joint contractures ‐ Severely reduced ROM or hip displacement that would prevent cycling ‐ Surgery, trauma, or fractures in last year ‐ Surgery/serial casting scheduled during trial ‐ Known cardiovascular/pulmonary conditions ‐ Uncontrolled epilepsy
Bryant et al.,^25^ UK	RCT	Special schools	35 Static bike: 11 Treadmill: 12 Con: 12	Bike: 14:4 (1:11) Treadmill: 13:6 (2:7) Con: 13:10 (2:4)	21:14 Bike 5:6 Treadmill 9:3 Con 7:4	IV/V 23:12 Bike IV/V 8:3 Treadmill IV/V 8:4	‐ Spinal or lower limb surgery in the previous year ‐ Cognitive or behavioural impairment preventing understanding or compliance with instructions
Colquitt et al.,^21^ USA	Randomized crossover	Home or school	12	14:6 (5:4) Range 7–24	4:8	I/II/III/IV/V 5:1:4:1:1^a^	‐ Surgery or botulinum neurotoxin A injections in previous 6 months ‐ Inability to understand/follow directions ‐ Epilepsy
Daly et al.,^23^ USA	Single‐subject ABAB	School	3	8:4; 10:8; 14:10	0:3	IV: 3	‐ Musculoskeletal or neurological surgical procedure in previous 6 months
Dodd et al.,^16^ Australia	Single‐subject AB	Community gymnasium	4	46:0 (6:5) Range 40–55	2:2	IV: 4^a^ (athetoid)	‐ Nil specified
Germain et al.,^37^ Australia	Single‐subject ABA	Facility (equipment located at Dreamfit community venue)	1	11:1	0:1	V: 1	‐ Severe pain ‐ Surgery in the previous year ‐ Osteoporosis or fractures in the previous year ‐ Hip migration percentage > 30% ‐ Spinal rods, fusions, metal implants, plates, screws, ventriculoperitoneal shunt, percutaneous endoscopic gastrostomy, stoma, baclofen pump, or other implants ‐ Hypermobility or deformity of lower limbs, atlanto‐axial instability, spondylolisthesis ‐ Uncontrolled epilepsy ‐ Presence of reflux or vomiting, hernia, retinal detachment, prolapse, haemorrhagic conditions, haemophilia ‐ Botulinum neurotoxin A in the previous 6 months ‐ Inability to reliably follow instructions
Hjalmarsson et al.,^38^ Sweden	Single‐group pre–post test	Indoor track and field facility	15	16 years Range: 9–29	7:8	I/II/III/IV 1:3:4:7	‐ Orthopaedic surgery ‐ Botulinum neurotoxin A injections during trial or 3 months previously ‐ Selective dorsal rhizotomy or intrathecal baclofen
Hüche Larsen et al.,^19^ Denmark	Non‐RCT	Community	15 Exp: 9 Con: 6	36 years (10 years) Exp 38 years (8 years) Con 34 years (15 years)	6:9 Exp 4:5 Con 2:4	III/IV/V 2:11:2 Exp III/IV/V 1:7:1	‐ Previous surgeries involving major restrictions to physical activity ‐ Uncontrolled seizures ‐ Unable to understand simple instructions
Moretto et al.,^39^ France	Pre–post case series	Community swimming pool	8	20 years (5:5) Range 13–28 years	2:6	I/II: 4 IV: 4^a^	‐ Intellectual disability ‐ Hearing impairment
Sansare et al.,^14^ USA	RCT	Home	36 FES cycle: 14 Vol cycle: 11 Con: 11	FES cycle 14:6 (2:5) Vol cycle 12:8 (2:1) Con 13:8 (2:11)	6:30 FES cycle 1:13 Vol cycle 3:8 Con 2:9	II/III/IV 12:11:13 FES cycle II/III/IV 6:3:5 Vol cycle II/III/IV 2:4:5 Con II/III/IV 4:4:3	‐ Leg orthopaedic surgery or traumatic fracture in 6 months; leg joint pain during cycling; leg joint instability or dislocation; leg stress fractures in 12 months ‐ Symptomatic or current diagnosis of cardiac disease; current pulmonary disease or asthma and taking oral steroids or hospitalized for an acute episode in previous 6 months ‐ Severe spasticity in legs; severely limited joint ROM or irreversible muscle contractures that prevented safe positioning on the cycle
Taylor et al.,^17^ Australia	Single‐group pre–post test	Community gymnasium	11	47:7 (8:2) Range 40–66	4:7	I 1 II 1 II/III 1^a^ III/IV 6^a^ IV/V 2^a^	‐ Participated in strength training in previous 3 months ‐ Not excluded if musculoskeletal impairments present (e.g. arthritis, joint deformity) as long as able to mobilize 10 m
Teixeira‐Machado et al.,^40^ Brazil	RCT	Community dance class	26 Exp: 13 Con: 13	Exp 18:0 (3:6) Con 17:1 (2:4)	Exp 7:6 Con 8:5	II/III/IV/V 9:8:7:2 Exp II/III/IV/V 6:3:3:1	‐ Cognitive or psychiatric impairments
Terada et al.,^18^ Japan	Single‐group pre–post test	Community care centre (residential living)	6	50:8 (8:11) Range 37:7–64:7	4:2	V: 6	‐ Severe communication difficulty ‐ Previous experience or involvement with sport or a special exercise programme ‐ History of cardiorespiratory disease ‐ Medications that could affect study results (e.g. beta blockers) ‐ Surgical history in the previous year
Vogtle et al.,^20^ USA	Repeated measures	Community day programme	26	42:4 (11:2) Range 23–63	16:10	I–III: 12^a^ IV–V: 14^a^	‐ Unable to follow directions or respond to questions on study instruments
Williams and Pountney,^24^ UK	Single‐subject ABA	Special school	11	12:7 (1:4) Range 11:2–14:10	10:1	IV: 8 V: 3	‐ Spinal or lower limb surgery in the previous year
Willoughby et al.,^26^ Australia	RCT	Special school	26 Exp 12 Con 14	10:10 (3:11) Range 5–18 Exp 10:5 (3:1) Con 11:2 (0:2)	Exp 6:6 Con 5:9	III: 8 IV: 18	‐ Needed physical assistance from another person to walk with their assistive device ‐ Concurrent medical condition such as severe cardiorespiratory disease or uncontrolled epilepsy that posed a safety risk ‐ Lower limb orthopaedic surgery or botulinum neurotoxin A injections in the previous 6 months
Wilson et al.,^22^ Australia	Single‐subject (multiple baseline)	University swimming pool (public access)	3	15:7 (0:7) Range 15–16	1:2	IV: 3	‐ Intellectual impairment affecting ability to follow instructions safely ‐ Severe physical impairment that individual would be physically unable to participate ‐ Significant dysphagia or oromotor impairments ‐ Recent serious musculoskeletal injury or illness ‐ Recent or scheduled surgery, e.g. single‐event multilevel surgery, correction of scoliosis ‐ Compromised skin integrity (ongoing) likely to affect safety of the individual, staff members, or members of the public ‐ Pain levels affecting ability to participate

Abbreviations: Con, control; Exp, experimental; FES, functional electrical stimulation; GMFCS, Gross Motor Function Classification System; RCT, randomized controlled trial; ROM, range of motion; Vol, volitional.

^a^ Estimated from non‐GMFCS classifications or from study description of gross motor function.

The research designs used in the reviewed studies were randomized controlled trials (*n* = 5), non‐randomized controlled trials (*n* = 1), randomized crossover trial (*n* = 1), single‐subject design (*n* = 5), pre–post test studies (*n* = 4), and a repeated measures study (*n* = 1). Study sample sizes were generally small: the largest trial enrolled 36 participants, 13 of whom had gross motor classifications in GMFCS level IV.^14^ The community settings for the studies included schools (*n* = 4), participants' homes (*n* = 3), gymnasia (*n* = 2), swimming pools (*n* = 2), an indoor track and field facility (*n* = 1), a day programme (*n* = 1), and other community settings (*n* = 2). Sixteen studies were completed in developed countries, and one in a developing country (Brazil).^15^ Most studies had been published in the previous 5 years (10 of 17 studies).

All included studies specified exclusion criteria, with one exception.^16^ Exclusion criteria were predominantly related to recent or upcoming medical management, specific medical comorbidities, and predicted ability to participate. Potential participants were often excluded if they had received surgery in the previous 6 to 12 months (*n* = 12 studies), botulinum neurotoxin A in the previous 3 to 6 months (*n* = 4 studies), or had sustained a fracture in the previous 6 to 12 months (*n* = 4 studies). Three studies excluded those who had upcoming scheduled surgery (e.g. single‐event multilevel surgery or correction of scoliosis) or another planned intervention (e.g. serial casting or injections of botulinum neurotoxin A).

Medical comorbidities relevant to exercise participation were exclusion criteria in 11 studies. Comorbidities included known cardiovascular or cardiorespiratory disease (*n* = 4 studies), with two studies specifying exclusion if the condition was severe (e.g. symptomatic, requiring medication, or hospitalization in the previous 6 months) and posed a safety risk during exercise training; medical comorbidities common in CP such as uncontrolled epilepsy (*n* = 4 studies), selective dorsal rhizotomy, intrathecal baclofen, or metal and other implants (e.g. spinal rods, plates, screws, ventriculoperitoneal shunt, percutaneous endoscopic gastrostomy, stoma); medications that could affect physiological responses to exercise (e.g. beta blockers); and other medical conditions (e.g. reflux, vomiting, hernia, retinal detachment, prolapse, haemorrhagic conditions, haemophilia). Eight studies specified musculoskeletal impairments or pain as exclusion criteria, particularly where it was believed it might prevent exercise participation such as severely reduced range of motion (joint contractures), joint instability, or dislocation, particularly of the hip (migration percentage > 30%), deformity of the lower limbs, atlanto‐axial instability, spondylolisthesis, hypermobility, and severe spasticity. However, one study did not exclude participants with musculoskeletal impairments as long as they were able to walk 10 m.^17^

Potential participants were also excluded on the basis of safety concerns, specifically where there was concern about their inability to reliably understand or follow directions (*n* = 8), or if they had a severe communication impairment (*n* = 1) or psychiatric impairment (*n* = 1). Two studies where the setting was a swimming pool specified exclusion criteria based on safety; these were ongoing compromised skin integrity, significant dysphagia or oromotor impairments, or a hearing impairment. A further two studies specifically targeted novice exercisers by excluding those with previous exercise experience.^17^, ^18^

Most study interventions targeted body functions (e.g. cardiorespiratory fitness or muscle strength; *n* = 10 studies) and/or activity limitations (e.g. gross motor function, transfers, walking; *n* = 11 studies). A smaller number of studies targeted participation in physical activity (*n* = 2 studies), improvements in pain and fatigue (*n* = 1 study), social skills (*n* = 1 study), or specific physical activity skills (e.g. swimming; *n* = 2 studies). The modes of exercise used were static, recumbent, or adaptive cycling (*n* = 5 studies), treadmill (*n* = 2 studies), weight machines/free weights (*n* = 2 studies), swimming (*n* = 2 studies), dance (*n* = 2 studies), rowing (*n* = 1 study), race running frame (*n* = 1 study), trampolining (*n* = 1 study), physical activities either embedded in daily life (*n* = 1 study) or performed as part of a group exercise class with weights and exercise bands (*n* = 1 study). The median intervention duration was 10 weeks (range 1–52 weeks), the median frequency was three times per week (range two to seven times per week), and the mean activity session duration was 30 minutes (range 25–90 minutes). Exercise was progressed during the intervention period in all studies where this was reported and overall attendance at scheduled sessions was high (Table 2).

**TABLE 2:** Characteristics of the interventions.

Reference	Intervention	Equipment	Supervision	Group vs individual	Aim	Duration (weeks)	Frequency (per week)	Session (minutes)	Exercise progression	Mean (SD) attendance
Armstrong et al.^36^	Aerobic FES and adapted cycling + HEP	Stationary motorized FES bike HEP: adapted bicycle; weighted vests	PT with or without allied health assistant and/or PT student HEP: parent or guardian	Individual	Improve capacity and function	8	3	60	Yes: increased resistance	91% HEP: 70% (38)
Bryant et al.^25^	Aerobic cycling or walking	Static bike or treadmill	PT	Individual	Improve gross motor function	6	3	Up to 30, including transfers	Yes: increased speed and/or duration as per ACSM guidelines	Bike: 14.6 (3.1); treadmill: 13.8 (4.2)
Colquitt et al.^21^	Aerobic rowing	Rowing ergometer	Research team: PG and UG kinesiology students	Individual	Increase power and function	6	3	25 (estimate)	Yes: during first 2 weeks of training	91.2%
Daly et al.^23^	Aerobic cycling	Adapted bicycle	Para‐professional educators under PT oversight	Individual	Improve cardiorespiratory fitness and gross motor function	16	5	30	Yes: increase in time and/or distance	85.7%
Dodd et al.^16^	Strength training	Weight machine or free weights	EP assisted by two staff with experience assisting people with physical disability	Group (four per group)	Improve muscle strength and performance of sit‐to‐stand	10	2	60 (estimate)	Yes: increased resistance as per ACSM guidelines	92.3% (6.9) Range 84–100%
Germain et al.^37^	Aerobic trampolining	Bungee trampoline	PT or PT student (under PT supervision)	Individual	Improve lower limb muscle strength, functional ability, provide moderate‐intensity activity	12	2	30	Yes: increased duration, cord height, and complexity of jumps	82.6%
Hjalmarsson et al.^38^	Aerobic race running	Three‐wheeled running bike with saddle and chest plate; no pedals	PT or sports science graduate	Group (5–15 per group)	Improve cardiorespiratory endurance	12	2	60 Mean 25 in motion (range 4–42)	Yes: increased duration/speed of short‐ and long‐distance intervals	Not reported
Hüche Larsen et al.^19^	Functional training (activities embedded in daily life)	Range of equipment to facilitate ADL	Individual: PT group: PT or caretaker from care home	Individual and small group	Improve gross motor function	12	3–5		Yes: reviewed performance against goals and progressed daily repetitions	Group sessions: 68%
Moretto et al.^39^	Aerobic swimming	Biofeedback swimming hand paddles	Swim coach (assumed)	Group (eight per group)	Improve stroke technique; increase aerobic fitness	1	7	55	Yes: increased distance and speed	‘Extremely high’
Sansare et al.^14^	Aerobic cycling with or without FES	Recumbent tricycle	PT who then trained parents	Individual	Improve cardiorespiratory fitness	8	3	Up to 30	Yes: increased duration	91.9%
Taylor et al.^17^	Strength training	Weight machines	EP plus accredited fitness instructor and two support staff with experience assisting people with physical disabilities	Group (five or six per group)	Improve muscle strength, mobility, speed, and sit‐to‐stand	10	2	Up to 60–90	Yes: increased resistance	94.9% (5.5)
Teixeira‐Machado et al.^40^	Aerobic dancing	Nil specific	Not reported (dance instructor assumed)	Group^a^	Improve physical fitness, balance and dexterity, functional independence, and social skills	12	2	60	Not reported	Not reported
Terada et al.^18^	Aerobic wheelchair dancing	Wheelchair dancer and ambulatory partner	‘Dance instructors’ (one from department of PT; one from graduate school of medicine; supervised by staff at community care centre)	Group (pairs)	Improve aerobic fitness	52	2	6–15	Not reported	Minimum frequency 2.0 (1.5) days/week; maximum frequency 3.1 (1.4) days/week
Vogtle et al.^20^	Group exercise	Arm ergometer, stationary bike (if able), weights, exercise bands, and balls	Fitness instructor with or without one or two assistants	Group (four to six per group)	Improve pain and fatigue; enhance physical activity	12	3	Up to 60	Not reported	81–100% except for two participants
Williams and Pountney^24^	Aerobic cycling	Adapted static bike with anterior and lateral trunk support, wrist supports, footplates, and ankle straps	PT and assistant	Individual	Improve function	6	3	Up to 30	Yes: increased duration and speed as per ACSM guidelines	88%; range 72–94% Sessions attended 16/18 (range 13–17)
Willoughby et al.^26^	Aerobic: treadmill training vs overground walking	Partial body weight supported; overground walking with assistive device	PT and/or oversight of trained assistant	Individual	Improve walking endurance, speed, and function	9	2	Up to 30	Yes: increased speed and reduced body weight support	Con: 14 (2) (range 10–17) Exp: 13 (2) (range 11–17)
Wilson et al.^22^	Aerobic swimming and land‐based resistance, aerobic, and neuromotor training	Resistance and aerobic training equipment	PT, EP, and swim coaches	Individual	Improve competitive swimming performance	32	1 initially; build to maximum 5	60	Yes: increased distance and resistance	Mean sessions per week (range) P1: 2.1 (1–3); 3.3 (2–5) P2: 1.7 (1–4); 2.3 (1–3) P3: 2.0 (1–3); 2.4 (2–3)

^a^ ‘Group’ was our interpretation of the methods.

Abbreviations: ACSM, American College of Sports Medicine; ADL, activities of daily living; Con, control; EP, exercise physiologist; Exp, experimental; FES, functional electrical stimulation; HEP, home exercise programme; P1–P3, participants 1–3; PG, postgraduate; PT, physiotherapist; UG, undergraduate.

All included studies implemented safety strategies as part of their intervention protocols (Table 3). Exercise was delivered as an individual programme (*n* = 10 studies), as a group programme (*n* = 6 studies), or with both group and individual components (*n* = 1 study).^19^ The intervention in 13 studies was either delivered by a health or exercise professional (e.g. physiotherapist or exercise physiologist) or overseen by a health professional. Most studies reported the involvement of an additional assistant(s) (e.g. student, family member, or volunteer) for implementation. In‐session physiological monitoring was a feature of the interventions in nine studies, most completed by heart rate monitoring. Only one study formally evaluated fatigue using a recognized fatigue survey (Pediatric Quality of Life Inventory multidimensional fatigue scale),^20^ and three studies included formal evaluation of pain.^20^, ^21^, ^22^ Other implemented safety strategies were pre‐exercise screening, a familiarization phase, supervision, education, and providing physical assistance (Table 3).

**TABLE 3:** Safety strategies.

Reference	Supervision	One‐on‐one supervision	Familiarization	Adapted equipment	Physiological monitoring during training	Physiological monitoring before and after training	Fatigue monitoring	Pre‐medical screen	Education	Physical assistance	Other
Armstrong et al.^36^	×	×	×	×	×		×^a^	×	×		× (Home visit)
Bryant et al.^25^	×	×		×						×	
Colquitt et al.^21^	×	×			×			×	×		× (Pain measure)
Daly et al.^23^	×	×		×	×						
Dodd et al.^16^	×									×	
Germain et al.^37^	×	×		×	×			×		×	
Hjalmarsson et al.^38^	×		×	×	×	×					
Hüche Larsen et al.^19^	×	×		×				×		×	
Moretto et al.^39^				×		×		×			
Sansare et al.^14^	×	×	×	×	×	×		×	×		
Taylor et al.^17^	×		×							×	
Teixeira‐Machado et al.^40^										×	
Terada et al.^18^	×					×				×	
Vogtle et al.^20^	×				×		×			×	× (Pain measure)
Williams and Pountney^24^	×	×		×	×			×		×	
Willoughby et al.^26^	×	×		×				×		×	
Wilson et al.^22^	×	×	×	×			×^b^	×			× (Pain measure)

^a^ Child‐initiated fatigue management strategy.

^b^ Training load, according to whole‐of‐life schedule, assessed weekly (from protocol^41^).

Attrition in trials was relatively low (0–23% where reported) (Table 4). Eleven studies explicitly reported whether adverse events occurred, and whether these events were related to physical activity (Table 4). Two of these studies reported no serious adverse events, injuries, or minor incidents.^23^, ^24^ Several studies reported minor incidents of soreness (*n* = 4 studies), fatigue (*n* = 4 studies), skin irritation (*n* = 2 studies), discomfort due to a harness that resolved by adjusting it and adding extra padding (*n* = 1 study), muscle spasms that resolved with stretching (*n* = 1 study), and a low‐speed non‐injurious fall (*n* = 1 study). A few adverse events that affected attendance or trial participation were reported. Five studies reported instances where participants described experiencing pain during training: in one study, two participants reported increased pain during training that did not require review by a physician;^21^ in four studies, participants reported pain that required them to cease exercising for a short period^17^ or resulted in them withdrawing from the trial.^20^, ^25^, ^26^

**TABLE 4:** Adverse reactions related to exercise and attrition.

Reference	Adverse reactions related to exercise	Attrition (%)
Armstrong et al.^36^	One × post‐training muscle soreness One × general muscle fatigue One × inner thigh chafing One × low‐speed fall from bike	0
Bryant et al.^25^	1 × reoccurrence of long‐standing hip pain (participant withdrew)	8.5
Colquitt et al.^21^	Increased pain in two participants during training; physician review not required	0
Daly et al.^23^	Nil	0
Dodd et al.^16^	Minor symptoms of soreness and fatigue No injuries or adverse events	0
Germain et al.^37^	Occasional soreness from harness (addressed by adjusting harness or applying extra padding) No serious adverse events	0
Hjalmarsson et al.^38^	Not reported	17
Hüche Larsen et al.^19^	Occasional minor muscle soreness, fatigue, and a few skin abrasions	6
Moretto et al.^39^	Not reported	0
Sansare et al.^14^	Not reported	7.6
Taylor et al.^17^	Minor symptoms of soreness and fatigue No major injuries or incidents reported One participant exacerbated pre‐existing back pain after leg press exercise but returned after 1 week without further problems One participant did not continue with leg press exercise after week 6 because of leg soreness while doing this exercise One participant withdrew at end of baseline phase as physical demands too difficult	9
Teixeira‐Machado et al.^40^	Not reported; ‘nil discontinued the intervention’	0
Terada et al.^18^	Not reported	0 (22 consented but only 6 participated)
Vogtle et al.^20^	Two x muscle spasms (wrist; quadriceps) One x low back pain in adult with dystonia (cushion sliding when exercising in power chair)—exercise cessation and withdrawal on physician advice	23
Williams and Pountney^24^	Nil post‐exercise muscle soreness; no signs of distress	18
Willoughby et al.^26^	Nil trips/falls; nil muscle/joint soreness during or after training One participant experienced back pain when walking in the assistive device and withdrew	21
Wilson et al.^22^	Not reported	0

## DISCUSSION

Our findings indicate that participation in physical activity interventions for most adolescents and adults with CP classified in GMFCS levels IV and V is safe and feasible. The findings endorse the capacity of people with more severe CP to participate in community‐based physical activity across different modes and in a variety of community venues. Their attendance at scheduled sessions was high (around 80% or more) and attrition relatively low (most commonly 0%). Furthermore, most interventions were designed to be delivered a median of three times a week, for a median of 10 weeks, for around 30 minutes per session; a dose that aligns with exercise and physical activity recommendations for people with CP.^27^

An important finding was the low number of adverse events reported and that most participants did not report an increase in pain or fatigue after physical activity. Only 4 of 160 participants (<2%) either withdrew from an exercise trial (three participants) or ceased exercise for a short period (one participant) because of pain. Previous studies have reported participants with CP describing a fear of injury, or new or increased pain or fatigue associated with physical activity, limiting or preventing their engagement. As many as 70% of adults with CP report living with chronic pain,^28^ and around 40% experience daily fatigue,^29^ so this hesitation to increase their physical activity is understandable. There is a high likelihood that anyone who is new to exercise will experience muscle soreness post‐training initially, and this was the case for those with CP classified in GMFCS levels IV and V; six studies reported ‘post‐training muscle soreness’ or ‘minor symptoms of soreness’ which quickly resolved without medical review and did not preclude continuing participation. Similarly, four studies reported some participants experiencing ‘minor symptoms of fatigue’ but this did not affect attendance or participation in the intervention. This low frequency of minor adverse events may suggest that factors other than the physiological response influence physical activity participation of people with complex CP. Adults with CP have a substantially greater risk of cardiovascular and respiratory disease than adults without CP^30^ and this risk is even further elevated in those with more complex disability.^31^ The review findings suggest physical activity, when implemented with specific safety strategies, is feasible to address the elevated health risks in people with complex CP.

Adolescents and adults with cognitive and communication impairments were frequently excluded from trials of physical activity interventions. Eight studies specified minimum cognitive or communication skills, or a requirement to be able to follow instructions as an inclusion criterion. Intellectual disability has been reported as being present in 45% of people with CP, increasing to around 83% in people with CP classified in GMFCS levels IV and V.^32^ Given the focus of this review was on identifying the implementation and safety of physical activity interventions in those with CP classified in GMFCS levels IV and V, potentially many people were excluded from the interventions evaluated. Similarly, the prevalence of epilepsy and other medical complexities is also highest in people with CP in GMFCS levels IV and V,^32^ and these were common exclusion criteria, even though physical activity is not contraindicated in epilepsy and many other medical conditions. In the limitations of the current literature (17 studies with 262 participants), much of which has systematically excluded those with significant cognitive and communication impairments, epilepsy, or other medical conditions, the risk of serious adverse events from community‐based exercise was very low (<2%). We recommend future studies ensure their samples are generalizable to the whole population of people with CP classified in GMFCS levels IV and V, to advance understanding of the benefits of physical activity in these groups and to ensure opportunities for participation in physical activity programmes.

In all studies, supervision or physical assistance was included as a component of the intervention, which meant the adolescent or adult with complex CP was rarely an independent exerciser. Overall, the nature of the supervision or physical assistance supports actually required was poorly reported, other than the professional background of the person providing the support (often a physiotherapist) and in some instances the staff to participant ratio. What remains unknown is whether supervision is a requirement for safe participation; that is, can the low numbers of adverse events reported be attributed to the specialist physical assistance and supervision provided or does the unspecified ‘risk’ remain low without assistance and supervision? This is particularly important as young people with CP have expressed their desire to exercise in community environments, where there may be few support staff available, and where staff may have less disability expertise than health professionals. We support that people with complex CP be afforded the dignity of risk in community settings; that is, the opportunity for personal growth through exploration and engagement in self‐determined, reasonable risk‐taking.^33^ Overall risk associated with exercise may be reduced through supervision, but the opportunity to take reasonable risk should not be precluded through exclusion. We advocate for future research to extend understanding in this area.

This review demonstrated successful access to physical activity programmes offered in educational facilities, community swimming pools and gymnasia, an indoor track and field facility, people's homes, and other community environments for adolescents and adults with complex CP. These facilities were generally accessible to the wider public. The physical environment has been reported as a major barrier to participation in community‐based physical activity for people with CP.^7^ A recent qualitative study^34^ reported ‘accessible and accommodating environments’ took many forms, including physical access, practical supports, reasonable accommodations/adjustments, and positive attitudes towards disability. Another recent study described the supports required for people with complex CP to successfully access a community‐based exercise facility, and highlighted significant additional time requirements from all stakeholders.^35^ Adapted equipment is frequently needed by people with disabilities which may limit their ability to engage without initial assistance or financial support to explore or implement equipment solutions or both. Options for sustainable community physical activity for adults with complex CP therefore requires not only physically accessible venues but also those where reasonable adjustments are made to the environment, and where staff and other participants are accepting, supportive, and not time‐limited.

Studies that included participants classified in GMFCS levels IV and V but were completed in a health setting or a non‐community venue such as a research laboratory were not included in this review. Additionally, grey literature was excluded. It was estimated that fewer than 10 participants (enrolled in three included studies) had not yet reached adolescence (i.e. were younger than 10 years of age at the time of enrolment); however, this represents fewer than 4% of all participants enrolled in the 17 included studies. Finally, all studies in this review reported time‐limited physical activity programmes. Any additional adverse events or risks associated with engaging in sustained physical activity programmes throughout the lifespan are unknown.

This study confirms that among previously sedentary adolescents and adults with CP with significant motor‐impairment, progressive physical activity can be performed in community settings with supervision, without excessive post‐exercise pain or fatigue, or serious adverse events. We posit that the known health benefits of physical activity, including limiting the development and progression of secondary multisystem disease, outweighs the small risk of adverse outcomes in this group. We encourage further physical‐activity‐based research in this population to maximize representation and generalizability of findings.

## Funding information

SC is supported through the Centre of Research Excellence by the National Health and Medical Research Council (grant APP1171758).

## Supporting information


**Appendix S1:** OVID Medline search strategy.

**Appendix S2:** Draft data charting template (adapted from JBI data extraction instrument and TIDieR checklist).

**Figure S1:** Identification of studies via databases.

## Data Availability

no data available
