# Supplementary material for: Community‐based physical activity interventions for adolescents and adults with complex cerebral palsy: A scoping review
Source: Dev Med Child Neurol. 2023 Apr 9;65(11):1451–63. doi: 10.1111/dmcn.15611 (PMC10952332; doi:10.1111/dmcn.15611)
Supplement: Supplementary file 1 — Appendix S1: OVID Medline search strategy. [file DMCN-65-1451-s001.docx]

| Search History (23) | |
| --- | --- |
| 1 | *cerebral palsy/ |
| 2 | (cerebral-pals* or spastic-dipleg* or spastic-hemipleg*).tw,kf. |
| 3 | 1 or 2 |
| 4 | Exercise Movement Techniques/ or exercise*.mp. or Exercise/ or Exercise therapy/ |
| 5 | “Physical activity”.mp. |
| 6 | Cycling.mp. |
| 7 | Aerobic.mp. |
| 8 | Hydrotherapy.mp. or *Hydrotherapy/ |
| 9 | Aquatic.mp. |
| 10 | Danc*.mp. or *Dance Therapy/ |
| 11 | Yoga.mp. or *Yoga/ |
| 12 | Pilates.mp. |
| 13 | “strength training”.mp. or *Resistance Training/ |
| 14 | Gym.mp. |
| 15 | “functional training”.mp. |
| 16 | “weight training”.mp. |
| 17 | Sport.mp. or *Sports/ |
| 18 | Exergaming.mp. or *Exergaming/ |
| 19 | “treadmill training”.mp. |
| 20 | Hippotherapy.mp. or Equine-Assisted Therapy/ |
| 21 | 4 or 5 or 6 or 7 or 8 or 9 or 10 or 11 or 12 or 13 or 14 or 15 or 16 or 17 or 18 or 19 or 20 |
| 22 | 3 and 21 |
| 23 | Limit 22 to (humans and “all child (0 to 18 years)” or “all adult (19 plus years)”)) |

Supplementary material 1: OVID Medline search strategy
