# Supplementary material for: Community‐based physical activity interventions for adolescents and adults with complex cerebral palsy: A scoping review
Source: Dev Med Child Neurol. 2023 Apr 9;65(11):1451–63. doi: 10.1111/dmcn.15611 (PMC10952332; doi:10.1111/dmcn.15611)
Supplement: Supplementary file 2 — Appendix S2: Draft data charting template (adapted from JBI data extraction instrument and TIDieR checklist). [file DMCN-65-1451-s002.docx]

Supplementary file 2

Draft data charting template (adapted from JBI data extraction instrument and TIDieR checklist)

Exercise for adolescents and adults with complex cerebral palsy: A protocol for a scoping review investigating the safety and implementation of physical activity programs.

**Review questions**

1. What are the safety strategies described when implementing community based physical activity interventions for people with complex cerebral palsy?

2. What are the safety outcomes (presence or not of adverse events)?

3. How are interventions implemented for people with complex cerebral palsy?

**Inclusion and exclusion criteria**

1. Population have a diagnosis of cerebral palsy (or those with cerebral palsy able to be extracted from mixed diagnoses group) and include participants with non-ambulant categories of GMFCS, Level IV and V. If ambulant (GMFCS Level I-III) participants were also included in the study, data pertaining to non-ambulant participants with CP must be able to be identified.

2. Mean age of 10 years or older (or data from this age range able to be extracted)

3. Study includes the delivery of a physical activity intervention

4. Study setting is in the community or home (not in a health facility)

5. Include all study designs (experimental and quasi-experimental)

6. Peer reviewed publication

| Bibliographic information | Authors |
| --- | --- |
|  | Title |
|  | Year |
|  | Journal |
|  | Volume and issue |
|  | Page numbers |
|  | DOI |
| Study characteristics | Country |
|  | Setting (eg home, school, community) |
|  | Study design and methods |
|  | Data sources |
|  | Who are the participants?  1) people with CP  2) mixed diagnoses |
|  | Number of participants |
|  | Number of participants with CP |
|  | Is data re participants with CP able to be extracted from larger cohort? |
|  | sex |
|  | Mean age and range |
|  | GMFCS Levels (or other indicators of function) |
|  | Comorbidities |
| Outcomes of interest | Intervention type (eg adapted bicycle, treadmill) |
|  | Intervention focus (eg fitness, strength) |
|  | Who delivered (eg physiotherapist, teacher) |
|  | Intervention dosage (minutes/days/weeks = total dosage) |
|  | Modifications (what, why, when, how) |
|  | Safety strategies (eg medical screening, physiological monitoring, adaptive equipment, hands on assistance) |
|  | Adverse reactions (safety incidents)  Reported yes/no  If yes, what is described (eg fall, muscle soreness, seizure) |
