# Supplementary material for: Community‐based physical activity interventions for adolescents and adults with complex cerebral palsy: A scoping review
Source: Dev Med Child Neurol. 2023 Apr 9;65(11):1451–63. doi: 10.1111/dmcn.15611 (PMC10952332; doi:10.1111/dmcn.15611)
Supplement: Supplementary file 3 — Figure S1: Identification of studies via databases. [file DMCN-65-1451-s003.pdf]

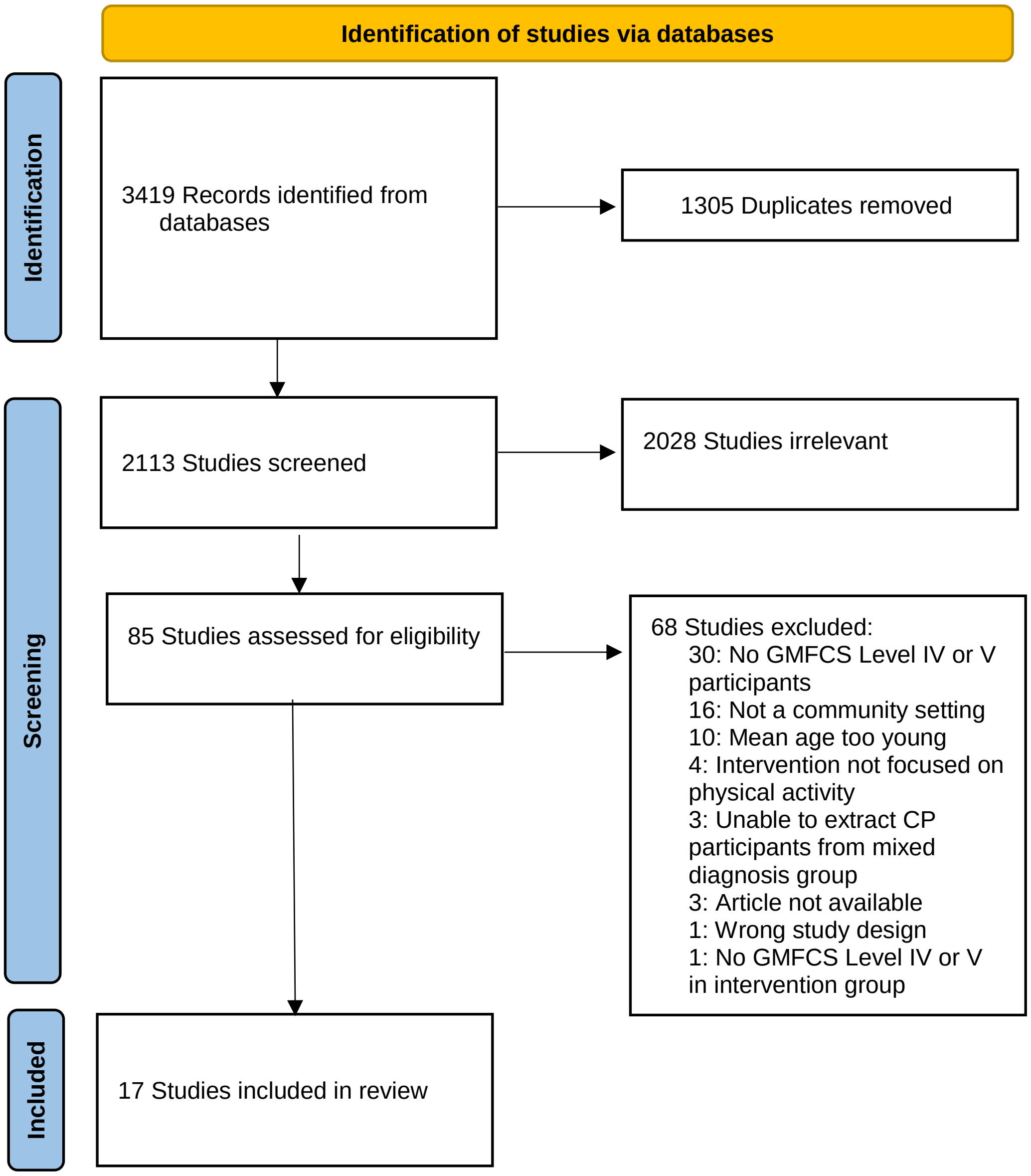

From: Page MJ, McKenzie JE, Bossuyt PM, Boutron I, Hoffmann TC, Mulrow CD, et al. The PRISMA 2020 statement: an updated guideline for reporting systematic reviews. BMJ 2021;372:n71. doi: 10.1136/bmj.n71

For more information, visit: http://www.prisma-statement.org/
